# Abstract of a Lecture on Comparative Odontology

**Published:** 1886-05

**Authors:** C. L. Ford

**Affiliations:** Professor of Anatomy and Physiology in the Medical and Dental Departments of the University of Michigan


### Abstract of a Lecture on Comparative Odontology.

Delivered before the Dental Association of Michigan on the Evening of March 16th, 1886.

BY C. L. FORD, M.D., PROFESSOR OF ANATOMY AND PHYSIOLOGY IN THE MEDICAL AND DENTAL DEPARTMENTS OF THE UNIVERSITY OF MICHIGAN.

*Mr. President and Gentlemen of the Association:*

When invited to address this assembly of dentists the first question was: What shall I say to men engaged in a calling like yours? To discuss the question of an advanced standard of dental education seems entirely superfluous, nor do I deem it worth while to take any subject of pathology or practice which will engage your deliberation while here.

Hence, I have decided to present some facts, by which I have endeavored to widen the range of knowledge of the dental students whom I have the honor to instruct.

In the brief time at my command I can only attempt a few facts and general principles, leaving the details largely for some other opportunity.

Any general survey of the animal kingdom soon shows that some animals develop teeth essentially uniform in size, shape, and manifest use; and this illustrates what is called *homodont* dentition.

A very superficial survey will soon show a large class of animals whose teeth vary very greatly in form, and this is called *heterodont* dentition, or differing teeth for different uses.

The student of comparative odontology soon discovers that some animals develop only one set of teeth which may or may not serve for the lifetime of the animal. *Monophyodont* describes this characteristic; developing teeth but once.

Further search soon demonstrates that another large class of animals develop teeth on the same plan as man, having two sets, adapted to differnent circumstances of food, etc. This is expressed by *diphyodont* dentition; two sets of teeth, or double dentition.

Again there are other animals that during their lifetime develop teeth continuously, in regular succession, or as they are removed by violence.

This mode is expressed by *polyphyodont* dentition, developing many teeth continuously. Under these three heads we may study *odontogeny*. But so large a field necessitates that I select some one or two prominent facts for more detail, so I choose,

### THE FORMS OF MAMMALIAN TEETH.

A general survey of these will show that they may be classed under four heads, as follows:

1, incisiform ; 2, cuspidate or caniniform ; 3, multi-cuspid, and 4, laminated.

The first, or incisiform, is well illustrated in the incisor teeth of the *rodentia*.

Here I remove an incisor of a beaver. Observe first its curve by which the pressure when used for gnawing purposes is so distributed that the pulp and its abundant vascular supply at its base are protected from results which would be likely to follow a like pressure on a tooth straight like a human incisor. The arrangement of these teeth, by which they occupy a curved canal beneath the molars, provides for great length as well as formid able size, and you observed the great difference in shape of the upper and lower incisors, where the latter illustrate the segment of a much larger circle than the upper teeth.

Any one who will study these teeth in various rodents will find admiration of the beautiful adaptation to such important uses increasing as his research extends.

The pulp within it continues to develop new material as the tooth is worn away, and when from any misfortune the upper and lower teeth no longer antagonize in use, the continued growth of the tooth assumes the curved form well illustrated by this tooth of a woodchuck, where manifestly a broken lower maxilla united in such a manner that the teeth no longer antagonize in a normal manner, and the ordinary abrasion was impossible.

The museums of the country contain exceedingly interesting exhibitions of the results of imperfect surgery in these unfortunate animals.

Observe also the marvelous beauty of the tooth structure, where, owing to the greater hardness of the convex side of the curve, the tooth wears in such a way as to be continuously sharp with a chisel-shaped cutting edge.

Indeed there seems to be a steadily increasing density from the inner to the outer part of the tooth as we study the structure of a transverse section of the tooth here displayed.

The second, or *cuspidate*, or canine form, is amply illustrated by the cuspid tooth in the skull of a bear or a dog, and abundantly illustrated in various animals.

The form is amply shown by this tooth of a mastodon, and by numerous other familiar teeth including our own molars.

The fourth or *laminated* form is a marvel of perfection for the hard and continuous service required during the lifetime of a horse, or an ox, in reducing the food supplied to a condition by which the nutritious material can be extracted.

A careful study of the teeth of these common vegetable feeders shows the perpendicular arrangement of laminæ of three degrees of density, viz: enamel, dentine, and cementum. An examination of the molar of the ox or the horse shows very readily how the surface is continuously rough and fitted to crush and cut the material furnishing the animal's food, and the peculiar arrangement and direction of the laminæ shows its adaptation for the necessary motion of the lower maxilla.

Any one who will take the trouble to examine with care, the manner in which these perpendicular plates or laminæ are curved, will see more than accident—a wise design in adapting means to ends in securing such important results from the nice arrangement of a few elements often arresting little attention.

This tooth of the elephant illustrates in a perfect manner the results of the laminæ of such varying density as I have here named.

Such brief consideration of these *four* varying *typical forms* of mammalian teeth may suffice to stimulate further examination in this interesting field.

I next ask attention to what I call *Mechanism of Mastication.* I here exhibit the articulation and motion of the lower maxilla in man. You see a shallow fossa in the temporal bone called glenoid, and a process or condyle of the lower maxilla, oval in form, fitted for motion in such shallow fossa.

Observe now the motion of this jaw of a bear, or of this dog, or of almost any carnivorous animal.

You observe only a vertical motion. The glenoid fossa is a transversely elongated groove, and the transversely elongated condyle, which you see just fits it, allows only vertical motion. This motion is most extensively illustrated in the animal kingdom. A glance at the arrangement of the teeth also shows that no lateral movement is possible.

If you will watch the mastication of the patient ox, you observe an entirely different motion, a lateral motion, a movement of the jaw sidewise. Now, look again at the laminæ of the teeth of the ox, and you may perhaps see an adaptation hitherto unnoticed, for cutting vegetable matter

In this connection, observe the relative width of the upper and lower maxillæ and see how perfectly the teeth are saved from injurious contact on the unused side in the ox or the horse, while minutely dividing the hay or grass.

Carefully adjust the upper and lower teeth of the ordinary ruminant, and you will see that in masticating, only lateral motion is possible, which this skull of the sheep abundantly illustrates.

Study also the articulation of the jaw of the horse, to illustrate the beauty of that plan to grind food by lateral motion, see the almost flat or level glenoid fossa, which allows the necessary motion laterally.

If now you study the articulation of a rodent animal, you will find an adaptation of glenoid fossa and condyle for a movement forward and backward. Here is an antero-posterior movement. Now, to increase your admiration for this peculiar arrangement, study the plan of the laminæ in the molar tooth of a rabbit or a beaver, where the prevailing direction is transverse.

Here then are illustrated the *three representative movements* of the lower maxilla in mastication:

1. A vertical movement.
2. A lateral movement.
3. A movement forward and backward, or as sometimes expressed, a to-and-fro movement.

I need hardly remind you that we imperfectly represent all these movements.

This hasty sketch must suffice for the present, but may perhaps awaken further investigation. Now, a few words as to the *number* and *location* of *mammalian* teeth.

A typical dental formula I define: "A number of teeth present in some animals and accepted as to number and variety of teeth by Odontologists, as a standard of comparison in describing mammalian dentition, conforming more or less to such standard."

The typical number of teeth is 44, written as follows:

$$\text{In}\ \frac{3-3}{3-3};\ \text{c}\ \frac{1-1}{1-1};\ \text{p m}\ \frac{4-4}{4-4};\ \text{m}\ \frac{3-3}{3-3}$$

The incisor teeth are located in the intermaxillary or premaxillary bone. In most animals this bone is distinct; in man it is early joined to the maxillary bone, usually leaving no trace of union.

The cuspid tooth is near the front of the upper maxilla, or close to the premaxillary suture. The premolars come out next and have successors.

The molars come in behind and have no predecessors. They develop as the jaw elongates, providing room for them as in man.

If the typical number is reduced, the incisors are retained from the centre laterally. Thus man represents the first and second incisor, the third is dropped or suppressed. The cuspid is suppressed in the female of some animals. In ruminants the upper incisors and cuspids are usually suppressed.

The premolars are usually suppressed from before backwards, thus man retains the third and fourth premolar, usually called bi-cuspid; but as premolars and molars are often nearly alike, as in the horse, the name is not based on the shape, but on location, where the language can be generally applicable, and not mislead by a name suggesting shape.

The molars are retained from before backwards. In man there seems a tendency to suppress the third molar, sometimes undeveloped.

I trust this hasty sketch may be the means of awakening an interest in comparative Odontology, for, as in man the student of Comparative Anatomy and Physiology gains a better understanding of himself. So by ranging over the field of Comparative Odontology, a man more fully comprehends the great work in a rich field opening before him, and is less apt to rest satisfied with a mere smattering or superficial knowledge of a subject so fitted, if wisely pursued, to lead the reflecting mind from beautiful results, to the Divine Architect who pre-arranged them.

With thanks for the close attention to these hasty utterances, I will not trespass further upon your time.

